# Effects of exercise on sleep spindles in Parkinson's disease

**DOI:** 10.3389/fresc.2022.952289

**Published:** 2022-08-11

**Authors:** Adeel Ali Memon, Corina Catiul, Zachary Irwin, Jennifer Pilkington, Raima A. Memon, Allen Joop, Kimberly H. Wood, Gary Cutter, Marcas Bamman, Svjetlana Miocinovic, Amy W. Amara

**Affiliations:** ^1^ Department of Neurology, University of Alabama at Birmingham, Birmingham, AL, United States; ^2^ Department of Neurosurgery, University of Alabama at Birmingham, Birmingham, AL, United States; ^3^ Department of Pathology, University of Alabama at Birmingham, Birmingham, AL, United States; ^4^ Department of Psychology, Samford University, Birmingham, AL, United States; ^5^ Department of Biostatistics, University of Alabama at Birmingham, Birmingham, AL, United States; ^6^ UAB Center for Exercise Medicine, University of Alabama at Birmingham, Birmingham, AL, United States; ^7^ Florida Institute for Human and Machine Cognition, Pensacola, FL, United States; ^8^ Department of Neurology, Emory University, Atlanta, GA, United States

**Keywords:** Parkinson’s disease, sleep spindles, exercise, sleep, cognition

## Abstract

**Background:**

In a randomized, controlled trial, we showed that high-intensity rehabilitation, combining resistance training and body-weight interval training, improves sleep efficiency in Parkinson's disease (PD). Quantitative sleep EEG (sleep qEEG) features, including sleep spindles, are altered in aging and in neurodegenerative disease.

**Objective:**

The objective of this post-hoc analysis was to determine the effects of exercise, in comparison to a sleep hygiene, no-exercise control group, on the quantitative characteristics of sleep spindle morphology in PD.

**Methods:**

We conducted an exploratory post-hoc analysis of 24 PD participants who were randomized to exercise (supervised 3 times/week for 16 weeks) versus 26 PD participants who were assigned to a sleep hygiene, no-exercise control group. At baseline and post-intervention, all participants completed memory testing and underwent polysomnography (PSG). PSG-derived sleep EEG central leads (C3 and C4) were manually inspected, with rejection of movement and electrical artifacts. Sleep spindle events were detected based on the following parameters: (1) frequency filter = 11–16 Hz, (2) event duration = 0.5–3 s, and (3) amplitude threshold 75% percentile. We then calculated spindle morphological features, including density and amplitude. These characteristics were computed and averaged over non-rapid eye movement (NREM) sleep stages N2 and N3 for the full night and separately for the first and second halves of the recording. Intervention effects on these features were analyzed using general linear models with group x time interaction. Significant interaction effects were evaluated for correlations with changes in performance in the memory domain.

**Results:**

A significant group x time interaction effect was observed for changes in sleep spindle density due to exercise compared to sleep hygiene control during N2 and N3 during the first half of the night, with a moderate effect size. This change in spindle density was positively correlated with changes in performance on memory testing in the exercise group.

**Conclusions:**

This study is the first to demonstrate that high-intensity exercise rehabilitation has a potential role in improving sleep spindle density in PD and leading to better cognitive performance in the memory domain. These findings represent a promising advance in the search for non-pharmacological treatments for this common and debilitating non-motor symptom.

## Introduction

Neurophysiological rhythms in the brain underlie the fundamental biological process of sleep (1). Sleep spindles are one such crucial oscillatory rhythm characteristic of non-rapid eye movement (NREM) sleep (2). This oscillation encompasses frequencies ranging from 11 to 16 Hz and originates in the thalamo-cortical loop (3). Sleep spindles are essential for declarative memory and sleep-related memory consolidation (3).

Although Parkinson's disease (PD) has historically been viewed primarily as a neurodegenerative motor disorder, we now have a clearer picture of non-motor manifestations of the disease (4–6). In fact, sleep disorders are frequently seen among individuals with PD and affect 74%–98% of patients (7, 8). Unfortunately, despite the profound impact of sleep dysfunction on PD, few pharmacological therapies have been shown to improve these symptoms, and the available treatments can have adverse side effects (9). In the absence of a biomarker, it has been difficult to find a treatment for cognitive dysfunction. However, a recent study by Latreille and colleagues found that sleep spindles are associated with the risk of cognitive decline in PD (10). Indeed, at the physiological level, spindles play an important role in the consolidation of memory (11).

The benefits of exercise on non-motor symptoms of PD are widely recognized (12, 13) as an exciting paradigm for nonpharmacological treatment. Indeed, a recent meta-analysis showed that exercise provided significant improvements in subjective sleep quality (14). Furthermore, our previous work and the only study in the literature to evaluate the effects of exercise on objective sleep outcomes showed an increase in sleep efficiency as measured by polysomnography (PSG) (15). However, to the best of our knowledge, no study has examined the effects of exercise on quantitative sleep EEG (qEEG) in PD (16).

With this objective in mind, this *post hoc* analysis leverages data collected in our recent clinical trial investigating the influence of exercise on objective sleep outcomes in PD (15) to address whether exercise also affects the microarchitecture of sleep spindles. This is a critical area of study due to the importance of spindles for memory consolidation, the high prevalence of both sleep and cognitive dysfunction in PD, and the limited treatment options available for these non-motor symptoms. We therefore sought to examine the effects of exercise on sleep spindle density and amplitude in a comprehensive manner. We hypothesized that exercise would increase spindle density and amplitude compared to a no-exercise control group and that changes in sleep spindles due to exercise would correlate with exercise-induced changes in memory performance.

## Methods

### Participants

**Figure 1:**
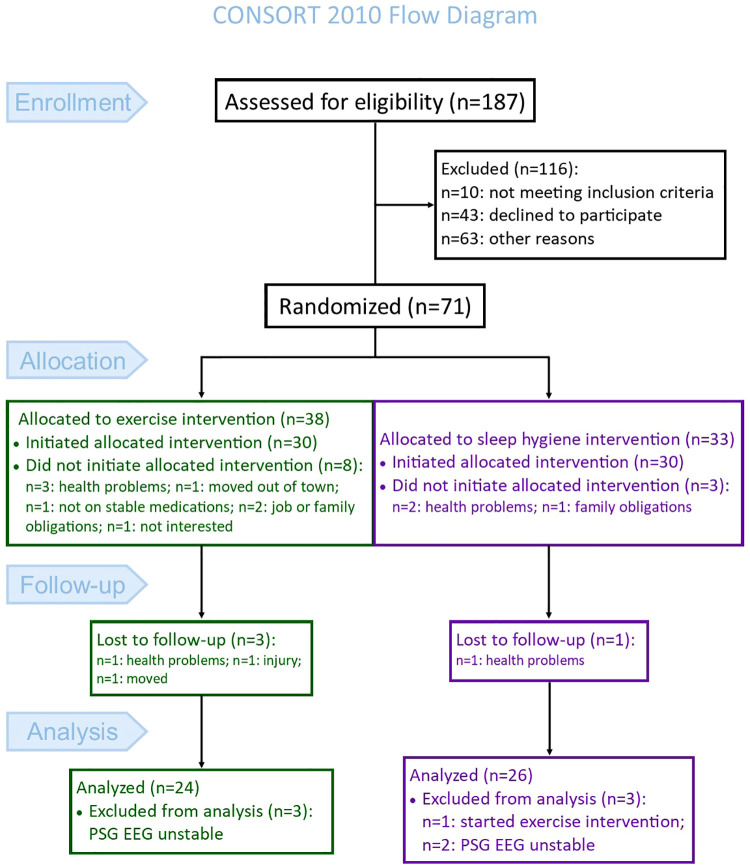
Consort flow diagram.

This study represents a *post hoc* analysis performed on polysomnography-acquired EEG collected during a randomized controlled trial (clinicaltrials.gov: NCT02593955). The primary objective of the parent trial was to investigate the impact of high-intensity exercise rehabilitation (EX), compared to a sleep hygiene no-exercise control (SH-C), on objective sleep outcomes in Parkinson's disease (15). The CONSORT flow diagram [Figure 1, adapted from (15)] shows the enrollment, group allocation, and follow-up of participants in the parent study. The analysis for the current study excluded five additional participants (*N* = 3 EX and *N* = 2 SH-C) because the sleep study file either at baseline or post-intervention were not usable for the qEEG analysis. Therefore, 50 participants (*N* = 24 EX and *N* = 26 SH-C) were included in the current analysis. Participants in this study were recruited from the Movement Disorders Center at the University of Alabama at Birmingham (UAB). Detailed eligibility criteria are previously reported (15); briefly, inclusion required: (1) diagnosis of PD based on the Movement Disorders Society's clinical criteria (17), (2) age >45 years, (3) on stable medications for at least four weeks prior to study entry, (4) Hoehn and Yahr stages 2–3, and (5) Montreal Cognitive Assessment (MoCA) ≥18. Exclusion criteria were: (1) atypical or secondary parkinsonism, (2) untreated sleep apnea, (3) inability to walk without a cane, (4) deep brain stimulator, or (5) meeting or exceeding the U.S. Health and Human Services physical activity guidelines (≥150 min/ week of moderate-intensity aerobic activity or 75 min/week of vigorous-intensity activity and muscle strengthening activities involving all muscle groups 2 or more days/week). Subjective sleep quality was not one of the entry criteria. The Institutional Review Board of UAB approved the study. Prior to participation, all participants signed a written informed consent.

### Intervention

#### Exercise group

Participants randomized to the EX group trained three times per week for 16 weeks at the UAB Center for Exercise Medicine under the supervision of experienced trainers certified by the American College of Sports Medicine. Sessions included resistance training (RT) and bodyweight functional mobility exercises to improve endurance, strength, power, and balance (15, 18), as previously described. Participants completed all sessions before 2 PM at the time they felt their PD medications were most effective. The intervention protocol was adapted from our previous dose-response optimization study in older adults (19, 20). In brief, the first four sessions consisted of a familiarization phase followed by a ramp-up phase, during which resistance training volume and intensity increased. Following ramp-up, RT intensity targeted 10-repetition maximum (10RM) in sessions 1 and 3 each week, while session 2 reduced resistance loads by 30% and maximized speed of movement during the concentric phase. The RT sessions included strength-building exercises (leg press, knee extension, chest press, overhead press, pull down); trunk exercises to improve postural stability (trunk extension/flexion); and body-weight exercises to improve balance and power (step-up, squat, jump squat, lunge, side lunge, push-up, assisted pull-up, assisted dip). In this manner, different muscle groups were targeted by alternating RT and bodyweight exercises. Heart rate (HR) was recorded with a Polar HR monitor throughout the sessions to determine short rest intervals between sets. Adherence was emphasized and the mean adherence to exercise was 92.2 ± 12.5% of sessions.

#### Sleep hygiene group

Participants randomized to the SH-C group received sleep hygiene counseling from a board-certified sleep medicine physician (AWA). During the counseling session, participants were given the opportunity to discuss their specific sleep complaints and were provided recommendations for improving sleep hygiene. Additionally, participants received written materials describing strategies for sleep relaxation and ways to improve insomnia. To enhance study engagement, SH-C participants were contacted every 4 weeks by telephone to address any questions related to sleep hygiene.

### Assessments

#### Polysomnography

Laboratory-based, supervised polysomnography (PSG) was performed at baseline and following the 16-week intervention. PSG was recorded with a 32-channel Natus Sandman Elite™ (Middleton, WI, USA), and included EEG, electrooculogram, submental and bilateral anterior tibial and extensor digitorum communis electromyograms, chest and abdominal polyvinylidene fluoride belts, airflow monitoring with thermocouple and nasal pressure, pulse oximetry and video. The EEG signals were digitized at a sampling rate of 512 Hz. EEG included frontal (F3, F4), central (C3, C4), and occipital (O1, O2) leads, all referenced to the contralateral mastoid. A certified sleep technician and a board-certified sleep physician (AWA) staged sleep in 30-second epochs according to the American Academy of Sleep Medicine's (AASM's) Manual for the Scoring of Sleep and Associated Events (21). Additionally, REM without atonia was scored according to the AASM scoring criteria (21). PSGs were labeled with a study code to allow blinding of group allocation during PSG interpretation.

#### Additional assessments

Participants were assessed for disease severity using the Movement Disorders Society-Unified Parkinson's Disease Rating Scale (MDS-UPDRS) (17). In addition, cognitive performance was evaluated in the memory domain. The tests in the memory domain included: (1) Hopkins Verbal Learning Test-Revised (HVLT-R) total recall and delayed recall; and (2) 10–36 Spatial Recall Test (10–36) immediate recall and delayed recall. Using the raw scores for the HVLT-R and 10–36, a normalized (z-) score was calculated based on normative values, controlling for age, race, and years of education, as appropriate for each test. Lastly, the individual test z-scores were averaged for each participant to determine the memory domain score.

### Quantitative sleep spindle morphological analysis

Preprocessing involved converting sleep EEG data into European Data Format (EDF); importing EDF into MATLAB (version R2020b); and inspecting each 30-s epoch for artifacts. The EEG evaluator (AAM) was blinded to the participant's group allocation. Because sleep spindle activity tends to be predominantly found in central leads during non-rapid eye movement sleep (NREM) (3), sleep spindle activity was analyzed in central leads and averaged over NREM stage 2 (N2) and stage 3 (N3). We visually examined the C3 channel for the entire PSG recording and detected electrical and movement artifacts. Whenever the C3 lead had continuous artifacts, C4 channels were used. Total artifact rejections pre-intervention included 3.9% of N2 and 1.0% of N3 for the EX group and 2.6% of N2 and 0.7% of N3 for the SH-C group. Post-intervention included: 2.8% of N2 and 0.8% of N3 for the EX group and 3.5% of N2 and 0.8% of N3 for the SH-C group.

A custom-made MATLAB script was used to detect sleep spindle events in the postprocessing step (3, 22). The following parameters were applied to the most artifact-free central channel: (1) frequency filter = 11–16 Hz, (2) amplitude threshold = 75th % percentile, (3) duration = 0.5–3 s. After that, the following sleep spindle characteristics were calculated and averaged over all N2 and N3 epochs of the whole night of PSG: (1) density (events/minute); and (2) amplitude (peak to peak, expressed in µV). In addition, we examined the spindle's characteristics over the first and second halves of the night because sleep spindle density has been shown to change over the course of the night in relation to elapsed sleep time (23).

### Statistical analysis

Statistical analysis was conducted using JMP Statistical Discovery Pro version 16.0. In the descriptive statistics, the Shapiro-Wilk test was used to identify the normality of all variables. We compared the demographic and polysomnographic characteristics between the EX and SH-C groups using Fischer's exact test for categorical variables and the independent sample two-tailed *t*-test for continuous variables. The main statistical methods for analyzing the intervention effects were general linear models with group x time interaction. Effect size was evaluated with Cohen's *d*. The significance level was set at *p *< 0.05. Due to the exploratory nature of our study, we did not correct for multiple comparisons and opted to tolerate possible Type I error rather than reject potential associations due to correction-induced Type II errors.

## Results

### Demographics, clinical and polysomnographic characteristics

In Table 1, the demographics and disease characteristics are summarized. There were no significant differences between the groups regarding age, sex, race, use of medications that impact sleep, disease duration, levodopa equivalent dose, or MDS UPDRS Parts I, II, III, or IV. In addition, there were no significant differences in baseline sleep architecture for the full night, first half, or second half of the night between the EX and the SH-C groups including sleep efficiency, total sleep time, sleep latency, wake after sleep onset, time and percent of each sleep stage, number of participants with REM sleep behavior disorder, apnea hypopnea index, or periodic limb movement index (all *p *> 0.05) (data not shown).

**Table 1:** Demographics and clinical characteristics.

Characteristics	Exercise group	Sleep hygiene group	*t/z/x* ^2^	*p*-value
*N*	24	26		
Age (years)				
Mean ± SD	65.7 ± 8.3	66.2 ± 5.1	0.28	0.78
Range	45–78	54–77		
Sex: *N* (%)				
Male	14 (58.3)	19 (73.1)	1.21	0.27
Female	10 (41.7)	7 (26.9)		
Race: *N* (%)				
Caucasian	22 (91.7)	25 (96.2)	0.45	0.5
African American	2 (8.3)	1 (3.9)		
Medications that affect sleep^a^: *N* (%)	8 (33.3)	7 (28.0)	0.164	0.69
Duration of Disease (DOD) (years)				
Median (IQR)	6.0 (2.3–8.0)	3.5 (1.0–8.0)	1	0.31
Levodopa Equivalent Dose (LED)				
Median (IQR)	620.0 (410.0–843.8)	545.0 (285.0–823.8)	0.58	0.56
MDS-UPDRS				
Part I				
Median (IQR)	7.0 (4.3–10.5)	9.0 (6.0–13.0)	−1	0.11
Part II				
Mean ± SD	10.8 ± 5.5	9.5 ± 5.2	−0.85	0.4
Range	0–24	1–23		
Part III				
Mean + SD	34.1 ± 12.9	29.0 ± 15.2	−1.28	0.21
Range	10–70	4–65		
Part IV				
Median (IQR)	3.0 (0.3–5.0)	3.0 (0.0–6.0)	0.18	0.86
Total				
Mean + SD	56.5 ± 18.2	51.6 ± 20.9	−0.88	0.38
Range	30–105	17–97		

Mean ± SD reported for normally distributed data. Median (IQR) reported for non-normally distributed data. MDS-UPDRS: Movement Disorders Society-Unified Parkinson's Disease Rating Scale.

^a^ Medications that affect sleep include benzodiazepines; non-benzodiazepine sedative hypnotics; narcotics; anti-psychotics; alpha-2 delta calcium channel ligands (gabapentin/pregabalin; melatonin; trazodone; oxybutynin; barbiturates.

### NREM quantitative sleep spindle analysis

**Figure 2:**
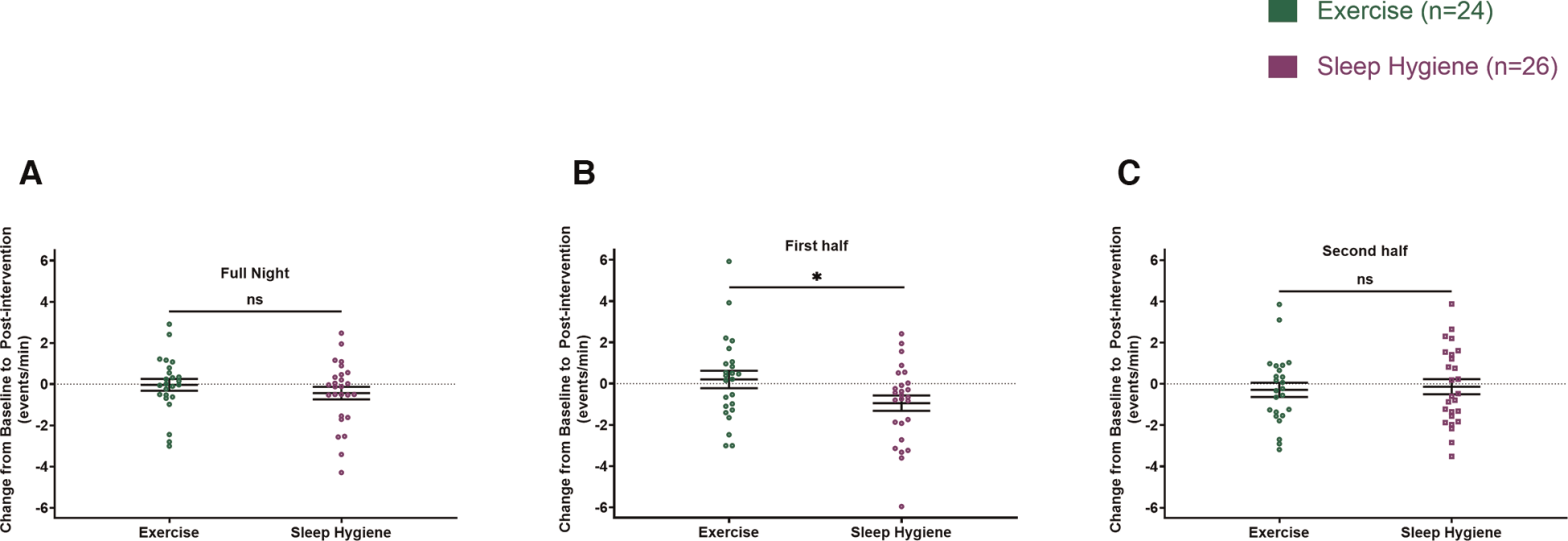
Exercise prevented the decrease in spindle density experienced during the first half of sleep by the sleep hygiene group (**B**). No significant changes in spindle density for the full night (**A**) and the second half of the sleep (**C**). In all panels, the figures are scatter plots, and error bars represent the mean and standard error of mean. ns: not significant.

**Figure 3:**
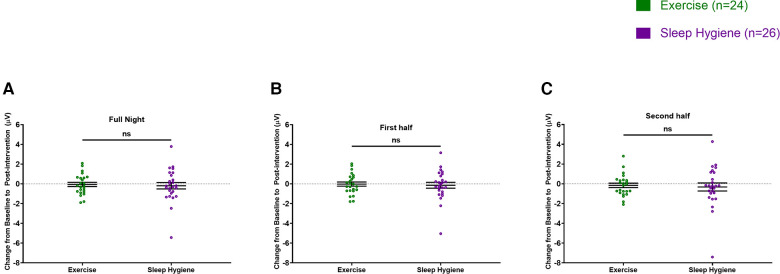
Spindle amplitude was not different between the two groups throughout the night (**A**), the first half of the sleep (**B**), or the second half of the sleep (**C**). In all panels, the figures are scatter plots, and error bars represent the mean and standard error of mean. ns: not significant.

There were no significant differences between changes in sleep spindle density or amplitude from baseline to post-intervention between the groups when evaluated for the entire night (Figures 2A, 3A). However, when analyzed separately for the first and second halves of the night, there was a significant decrease in spindle density in the SH-C group (*F* = 6.40, *p* = 0.0181) in the first half of the night and exercise attenuated this decrease in spindle density experienced by the SH-C group (i.e., a significant group x time interaction; *F  *= 4.19, *p* = 0.046; *d *= 0.58) (Figure 2B). No significant differences were observed in spindle density or amplitude for the second half of the night (Figures 2C, 3C).

### Memory performance and spindle analysis

**Figure 4:**
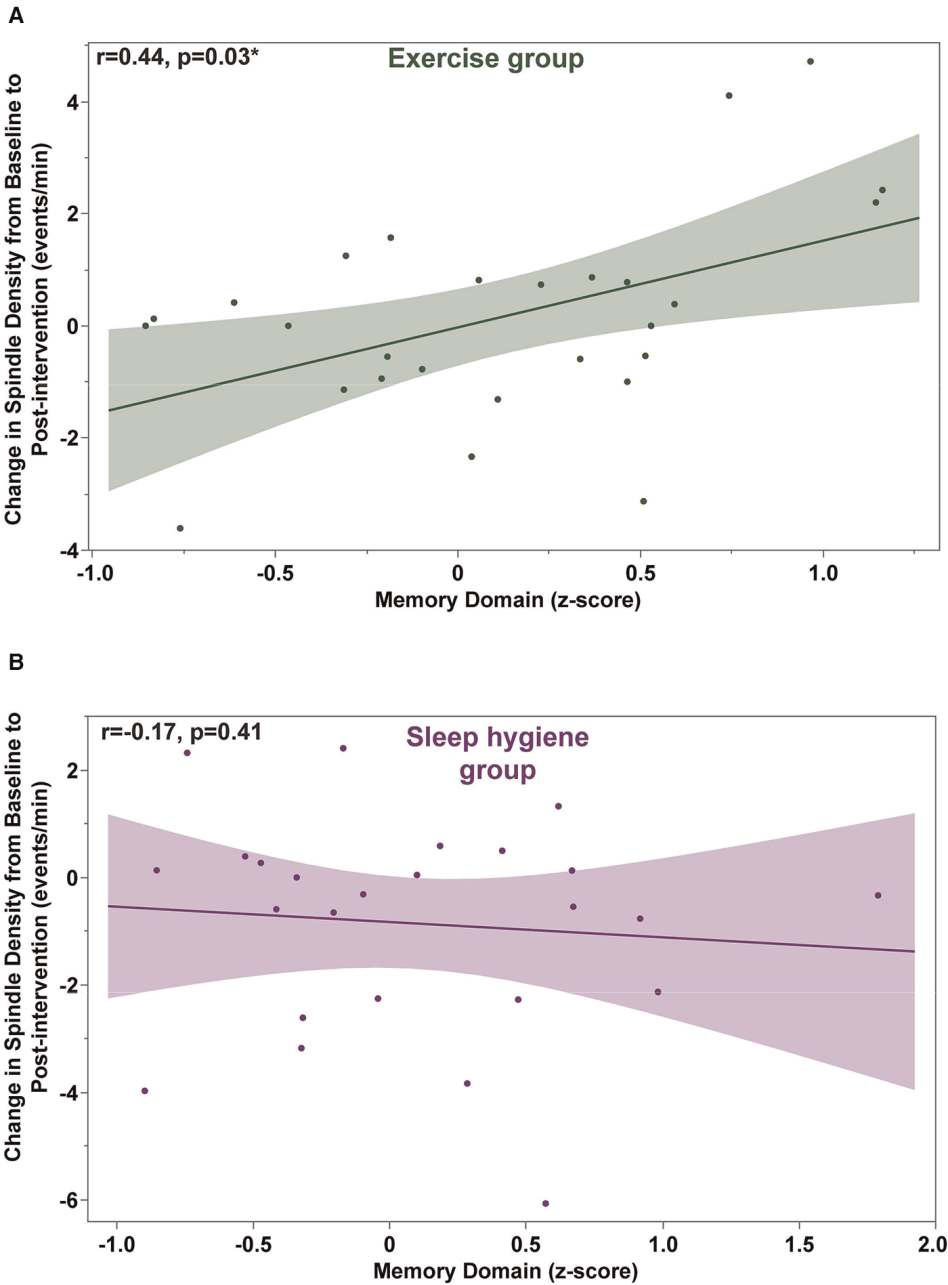
Exercise-induced changes in spindle density during the first half of the night are significantly correlated with changes in the memory domain scores (**A**). No significant correlations were found in the sleep hygiene group (**B**).

Next, we evaluated whether the beneficial effects of exercise in preventing decrease in spindle density were associated with better memory performance. The change in sleep spindle density in the EX-group was correlated with the change in the memory domain due to exercise (*r *= 0.42, *p *= 0.0413). A detailed analysis revealed that this effect was primarily driven by changes during the first half of the night (Figure 4). There was no significant correlation between the change in sleep density score in the SH-C group and the change in the memory domain. There was no significant correlation between the change scores in spindle amplitude and the change scores in the memory domain for either group (data not shown).

## Discussion

In this *post hoc* analysis of polysomnography-derived EEG from a randomized controlled clinical trial, exercise rehabilitation counteracted the decline in spindle density during NREM in the first half of the night when compared with a no-exercise control group with a moderate effect size (*d *= 0.58). In addition, exercise-induced changes in spindle density were positively correlated with better cognitive performance in the memory domain. Although this investigation is exploratory, the findings inform directions for future larger studies with investigation of exercise-induced changes in sleep spindles as the primary outcome. Because available pharmacological treatments for sleep and cognitive dysfunction are ineffective or have intolerable side effects (24), the current study represents a potential step towards identifying mechanisms of nonpharmacological interventions on sleep and cognition in PD.

Exercise has been shown to significantly improve subjective sleep quality in people with PD (14). The only study in the literature that evaluated the effects of exercise on objective sleep outcomes demonstrated an increase in sleep efficiency as measured by PSG (15). However, there was no change in the amount of N2 sleep. We posited that one possible reason for the lack of exercise-induced changes in stage N2 might be the semi-quantitative nature of the sleep-stage scoring system. In fact, for more than 50 years, since 1968, sleep has been evaluated by applying standardized scoring criteria to electroencephalograms and electromyography recordings developed by Rechtshanffen and Kales (25) and later modified by the American Academy of Sleep Medicine. The limitation of qualitative sleep staging is that stage N2 is scored only if one or more trains of K-complexes or sleep spindles can be observed during a 30-second epoch. Moreover, qualitative staging does not quantify the number of spindles, which have been shown to correlate with markers of cognitive performance (23). As PD involves the thalamocortical circuits in which spindles are generated (3), and spindle density predicts the development of PD dementia (10), we investigated the effects of exercise on spindle density. We found that exercise significantly prevented the decrease in spindle density experienced by the SH-C group during the first half of sleep. The rationale behind evaluating NREM separately during the first and second halves of the night is that physiologically, NREM sleep dominates during the first half of the night, whereas REM sleep dominates during the latter half (26). In addition, the first half of the night appears to have a differential effect on spindle activity, based on previous studies. For example, in a study by Dang-vu and colleagues, predictive relationships between spindle density and stress were only observed during the early stages of NREM sleep (27). Furthermore, Lopez and colleagues found that teenagers who were depressed had lower spindle density during the third and fourth stages of their NREM sleep cycle than those who were not depressed (28). It may be possible that in PD the beneficial effects of exercise are modulating NREM during the first half of the night. Also, the reason for the decrease in spindle density in the no-exercise group could represent natural progression of disease or may be related to other causes. However, the expected longitudinal change in spindle density is not well established in PD, so will need additional study. To our knowledge, no prior study has investigated the effects of exercise on sleep qEEG in neurodegenerative disease (16).

There are many proposed mechanisms to explain ways in which exercise may affect sleep, including exercise-induced decreases in inflammation, changes in core body temperature, changes in neurotransmitters that affect sleep, alterations in growth hormone or brain-derived neurotrophic factor (BDNF), changes in heart rate variability, and changes in autonomic function (29–32). In the parent clinical trial, sleep efficiency was improved in the exercise group, which may have resulted in part from increased spindle density since spindles play a crucial role in maintaining and sustaining sleep (2, 3). The mechanism underlying the exercise-induced alterations in sleep spindles cannot be determined from the current study but should be explored in future studies.

The contribution of sleep spindles to cognitive function is well established in physiological aging, mild cognitive impairment and neurodegenerative diseases (10, 33–36). Furthermore, a longitudinal study by Latreille and colleagues has demonstrated that baseline spindle density is also predictive of the development of dementia in patients with PD (10). Therefore, we evaluated the relationship between exercise-induced changes in spindles and changes in cognition. Change scores in spindle density were positively correlated with improved performance in the memory domain in the exercise group but not in the sleep hygiene group. Even though these findings are exploratory, given the prevalence of cognitive decline in PD, and the limited available treatment options, this is an exciting new development that should be further examined in future research.

Sleep spindle amplitude represents synaptic strength and synchronization of excitatory postsynaptic potentials and prior work has shown that PD patients have reduced spindle amplitude compared to controls (10). In this cohort, exercise did not significantly change sleep spindle amplitude. Therefore, it is not surprising that neither EX or SH-C showed correlations between changes in spindle amplitude and memory performance. It is necessary to conduct additional studies to confirm these findings; however, it may be that exercise-induced changes are more sensitive to spindle density than to amplitude.

There are several strengths to this study, including the utilization of polysomnography-derived EEG collected during a randomized, controlled clinical trial and the use of automated qEEG analysis methods. There are also several important limitations, including the *post hoc*, exploratory nature of the analysis. Additionally, there was no correction for multiple comparisons because one purpose of this analysis was to generate hypotheses for future research. However, it is important to be aware of this when interpreting the findings.

In conclusion, to our knowledge, the present study is the first to examine the effects of exercise on quantitative sleep spindle morphology in PD. Exercise rehabilitation may have potential benefits in preventing a decline in spindle density during the first half of the night compared to a no-exercise control group. Further, the effects of exercise on spindle density were positively correlated with better memory performance. Due to the exploratory nature of this study, future studies should examine the potential beneficial role of rehabilitation-induced changes in spindle density and cognition, given the high prevalence of this nonmotor manifestation and the limited treatment options available. These findings have important therapeutic implications and represent an exciting advance in the search for non-pharmacological treatments for this common and debilitating non-motor symptom.

## Data Availability

The original contributions presented in the study are included in the article/Suplementary Material, further inquiries can be directed to the corresponding author/s.
